# Thermopressure Coupling Effect Mimicking Natural Graphite Formation to Enhance the Storage K–Ion Performance of Carbonaceous Heterostructures

**DOI:** 10.34133/research.0092

**Published:** 2023-03-27

**Authors:** Tianyi Ji, Xiaoxu Liu, Hui Wang, Yunli Shi, Yang Li, Man Zhang, Junqi Li, Hui Liu, Ze Xiang Shen

**Affiliations:** ^1^ Shaanxi Key Laboratory of Green Preparation and Functionalization for Inorganic Materials, School of Material Science and Engineering, Shaanxi University of Science and Technology, Xi’an 710021, Shaanxi, China.; ^2^ Division of Physics and Applied Physics, School of Physical and Mathematical Sciences, Nanyang Technological University, Singapore 637371, Singapore.

## Abstract

Borrowing from natural mechanisms for material design can lead to functional mimicry and improvement. Inspired by graphite formation, a thermopressure coupling strategy under micropressure (<400 Pa) is applied to prepare carbon anodes. A thermopressure response is discovered based on the cellulose precursor. Here, homologous graphene quantum dot/hard carbon (GQD/HC) heterostructures are synthesized. Under 181.4 Pa and 1,200 °C, the product shows a capacity of 310 mAh g^−1^, while the capacity of the direct carbonization product is only 120 mAh g^−1^. Prominently, the GQD/HC heterostructure displays marked mechanical strength and flexibility. The experimental and theoretical results illustrate the ion and electron transfer, coordination environment, and electronic states in the GQD/HC heterostructure and elaborate on the origin of the enhanced performance. The thermopressure coupling under micropressure mimics graphite formation, but the heterostructure has better properties than traditional carbon materials. Additionally, micropressure injects new vitality into material research.

## Introduction

Currently, the prices of lithium salt have increased significantly because of the high demand for electrochemical energy storage [1,2]. K–ion batteries (KIBs) have been appreciated for several years because the K element has similar properties to and more abundant resources than lithium [3,4]. Considering the commercialization of graphite anodes, many studies on graphite for K–ion storage have been reported. Although graphite has a low voltage plateau (<0.3 V), the performance, such as capacity and rate, is not satisfactory because of the large K–ion radius [5]. Hence, the focus turns to structure regulation strategies of various carbon materials to improve the comprehensive performance of K–ion storage. First, many studies focus on the crystal structure of carbon materials. For example, few-layer graphene (with decreased layers) and expanded graphite (with large interlayer spacing), as a type of graphite-like carbon, also show a low plateau, but it is difficult to consider both capacity and rate [6,7]. In addition, while many turbostratic carbon materials (such as hard or soft carbon) have high capacity and rate performance, they suffer from unbalanced plateaus and capacities caused by their amorphous structures [8,9]. Second, defect engineering has been widely adopted to adjust the physicochemical properties of carbon nanomaterials. For instance, the heteroatom-doping strategy significantly increases the number of adsorption sites of K ions and the conductivity [10–12], and the pore structure design usually increases the ion diffusion ability by offering adequate pathways [13–15]. However, defect engineering usually results in a wide working voltage, which is gradually dominated by adsorption behavior [16]. Moreover, building carbon composites provides an idea to improve the performance [17,18]. Recently, a carbon dot/reduced graphene oxide composite was reported and exhibited synergistic advantages. Composites based on hard carbon (HC), soft carbon, or graphite have also been successively reported and have realized the complementary advantages of the 2 components [19,20]. Considering the composite strategies, this method is mostly biased toward physical composites, which is not conducive to mechanical properties and flexibility to some extent. To date, a major challenge is to find a design strategy that improves the comprehensive performance and simplifies the preparation process to meet the diverse applications of carbon materials.

Nature has been a source of inspiration for the design of high-performance engineering materials [21]. During geological processes, organic matter gradually develops into highly crystalline graphite at high temperature and pressure (at the level of gigapascal) [22,23]. In the formation of natural graphite, the thermopressure coupling effect is remarkable. For instance, the pore walls of graphite intermediates are made of straight and perfect layers when thermopressure coupling is applied [24]. Meanwhile, under appropriate temperature and pressure, the degree of graphitization, crystal, and electronic structures of carbon materials are significantly altered [24–26]. Although the carbon materials obtained by thermopressure coupling under high pressure do not show satisfactory K–ion storage performance, there is still substantial room for improvement. Until then, some important issues must be considered: (a) How to determine the temperature and pressure to improve the overall performance and ensure the possibility of large-scale application, (b) how to analyze the carbonization rule based on the thermopressure coupling effect, and (c) how to establish the relationship between K–ion storage properties and carbon products under thermopressure coupling.

In this article, by mimicking the thermopressure coupling conditions of graphite formation, a thermopressure coupling strategy under micropressure (<400 Pa) is applied to prepare carbon anodes. A novel thermopressure response is discovered based on a cellulose precursor, i.e., the micropressure restricts the escape of gaseous molecules at high temperatures; then, homologous graphene quantum dot/HC (GQD/HC) heterostructures are synthesized after carbonization of primary coke and secondary coke. The micropressure regulates the orientation of the carbon layers and results in heterostructures. Most notably, the heterostructure has remarkable mechanical strength, which is rare in amorphous carbon materials prepared at high temperatures. When the heterojunction that was prepared at 181.4 Pa and 1,200 °C is applied to the KIB anode, it shows a capacity of 310 mAh g^−1^ and an initial coulombic efficiency (ICE) of 67%, while the capacity and ICE of the direct carbonization product are only 120 mAh g^−1^ and 34%, respectively. Combined with electrochemical testing, synchrotron technology, and theoretical calculations, it is proven that the enhanced K–ion storage performance stems from the unique structure of the GQD/HC heterostructure. The thermopressure coupling effect under micropressure mimics the formation process of graphite, but the comprehensive properties of the heterostructure exceed those of graphite. In particular, micropressure is easy to apply on a large scale, while providing rich opportunities for material research.

## Results and Discussion

**Fig. 1.**
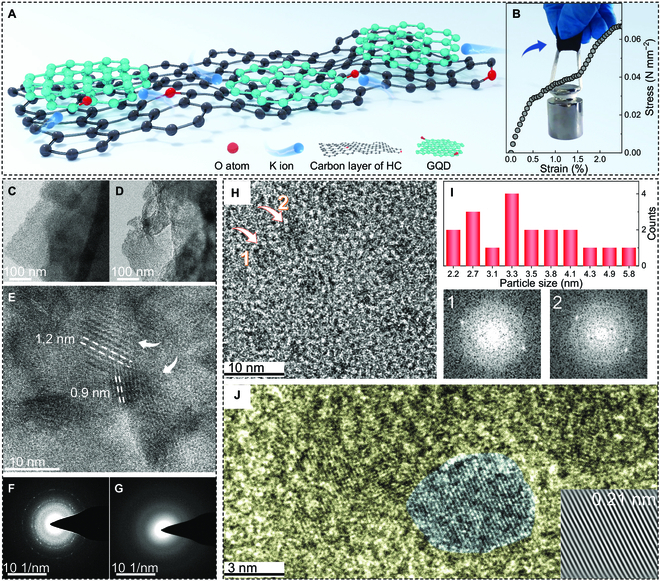
Microstructure analysis and mechanical behavior. (A) Schematic illustration of carbon materials prepared by the thermopressure coupling strategy under micropressure. (B) Stress–strain curve and a digital photo of GQD/HC-1200. (C and D) TEM images of GQD/HC-1200 and HC-1200, respectively. (E) High-resolution TEM (HRTEM) of GQD/HC-1200 with moiré fringes. (F and G) SAED patterns of GQD/HC-1200 and HC-1200, respectively. (H) HRTEM of GQD in GQD/HC-1200. (I) Size distribution statistics of GQD and fast Fourier transform patterns of corresponding areas. (J) HRTEM of GQD/HC-1200 and lattice spacing.

First, the SEM image of GQD/HC-1200 shows hollow fiber (Fig. S2A and B). The HC-1200 also shows hollow fiber morphology, but its degree of fragmentation is large (Fig. S2C and D). In the TEM images of 2 samples, the carbon layer of GQD/HC-1200 is intact with no obvious pores (Fig. 1C), while the carbon layer without pressure is rough and porous (Fig. 1D). Therefore, the mechanical strength of GQD/HC-1200 may change. Interestingly, the distinct moiré fringes are observed in the high-magnification TEM image of GQD/HC-1200 (Fig. 1E). Moiré fringes are common in 2-dimensional materials of few layers combined with the van der Waals force [27]. Hence, GQDs are formed in GQD/HC-1200, and the interlayer coupling is weak among GQD and HC based on the van der Waals force. Therefore, they could be stacked in any combination to form heterostructures without the restriction of lattice matching, making it possible to construct moiré superlattices by turning angles and other ways [28]. The moiré period observed in Fig. 1E is within ~10 nm, which indicates the small lattice mismatch between the top and bottom layers. This observation demonstrates that the GQDs and HC constitute a heterostructure as 2 independent components based on the thermopressure coupling effect under micropressure.

Furthermore, the selected area electron diffraction (SAED) pattern of GQD/HC-1200 shows obvious diffraction rings and spots (Fig. 1F). On the contrary, the HC-1200 only shows diffraction rings (Fig. 1G), which confirms that GQD with good crystallinity is formed with the assistance of micropressure. Hence, as shown in Fig. 1A, the typical HC is derived from the cotton precursor, and the GQDs are distributed in the carbon layers of HC. Both structures are stacked by graphene layers, which can form the heterostructure based on the van der Waals force. However, there are differences between them in the crystallinity, such as grain size and interlayer spacing. Notably, the formation of GQD improves the carbon yield and avoids the large-scale destruction of the carbon skeleton. Therefore, GQD/HC-1200 exhibits obvious mechanical strength (Fig. 1B), whose stress–strain curve shows the stress yield phenomenon. At the same time, the material can easily pull up 200-g weight, indicating significant mechanical properties. However, HC-1200 cannot pull up the lighter weight at all (see Fig. S3 and Movies S1 and S2). In contrast, the natural graphite minerals formed at high pressure are fragile. This work draws on the idea of natural graphite synthesis, but a carbon material with better mechanical properties is obtained under micropressure.

Then, Fig. 1H shows a clear distribution of GQD. According to the size analysis of GQD in several TEM images (Fig. S4), the size distribution is mostly less than 5 nm, and most of them are about 3 nm (Fig. 1I). The fast Fourier transform of 2 GQD regions labeled 1 and 2 in Fig. 1H shows that there are obvious diffraction spots, indicating the high crystallinity of GQD. After further magnification (Fig. 1J), there is highly crystalline GQD covered on disordered HC, in which the lattice fringe of GQD is 0.21 nm, corresponding to the (100) plane of graphite [29]. In addition, the heat treatment temperature also has a regular effect on the heterojunction structure (see details in Fig. S5). From the SEM, TEM, and SAED patterns, it can be found that the morphology of the carbon material prepared under micropressure changes greatly. Micropressure helps to improve the integrity of the carbon skeleton and form GQD, which significantly enhances mechanical strength.

**Fig. 2.**
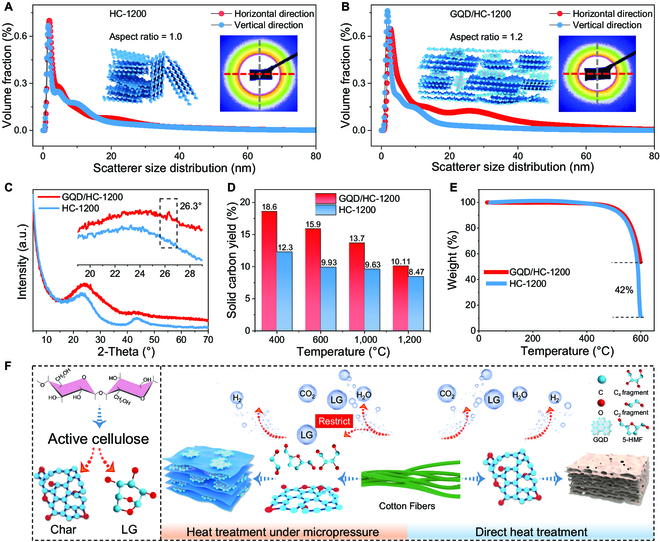
Structural characterization and preparation mechanism of 2 samples. (A and B) Integration results of synchrotron SAXS data and original scattering patterns. (C) XRD spectra. (D) Solid carbon yield. (E) Thermogravimetric curves. (F) Main pyrolysis process of cellulose and formation mechanism of GQD. a.u., arbitrary units.

Next, SAXS was used to explore the structural changes induced by thermopressure coupling. As shown in the illustrations (Fig. 2A and B), HC-1200 shows a symmetric circular SAXS scattering image (the aspect ratio is 1.0), but there is an obvious asymmetrical elliptical shape (the aspect ratio is 1.2) for GQD/HC-1200, indicating the orderly stack of carbon layers. After continuing to integrate the SAXS 2-dimensional scattering image along the horizontal and vertical directions, the 1-dimensional data of synchrotron SAXS were obtained (Fig. S6). The tangent-by-tangent method was used to analyze the volume distribution of the pore size (point plot in Fig. 2A and B) [30].

In Fig. 2A, the pore size distribution of the HC-1200 sample in 2 directions shows similar results with a wide distribution within 30 nm, indicating high disorder and corresponding to the TEM results (Fig. S7). For GQD/HC-1200, there is a obvious difference along the horizontal and vertical direction (Fig. 2B). The unique mesoporous structure is around 26 nm displayed in the horizontal direction, suggesting that the orientation growth of the carbon layer under micropressure affects the mesoporous structure. Meanwhile, the curves in both directions show peaks around 5 and 9 nm, corresponding to the micropore structure. The above analysis is also confirmed by the pore size analysis and the TEM results (Fig. S8). On the basis of the results of synchrotron SAXS, TEM, and N_2_ adsorption–desorption, the structural illustrations in Fig. 2A and B describe that the arrangement of the carbon layers will be limited by micropressure, which prevents the random growth of the carbon layers during heating, so the structural orientation appears. During oriented growth and stacking of carbon layers, the mesoporous structure also has orientation characteristics, which leads to the difference in synchrotron SAXS results. At the same time, compared to disordered stacking, the oriented arrangement of carbon layers also contributes to the improvement of mechanical strength.

Next, both samples show obvious wide peaks in the XRD pattern (Fig. 2C), reflecting the amorphous state of HC. However, GQD/HC-1200 has an obvious diffraction peak at 26.3°, corresponding to the (002) plane of GQD. Furthermore, the grain size of the carbon layers was calculated for different samples according to XRD data (Fig. S9 and Tables S1 and S2). As the temperature increases, the interlayer spacing *d*_002_ gradually decreases and the grain size (*L_a_* and *L_c_*) gradually increases for the carbon layers of 3 samples with micropressure. On the other hand, *L_a_* in GQD/HC-1200 is significantly larger than that in HC-1200, while *L_c_* is slightly smaller. As the pressure increases, the *d*_002_ gradually decreases and *L_a_* gradually increases, but *L_c_* is in a decreasing trend. XRD results indicate that micropressure inhibits the growth along the *c* axis of carbon layers and promotes the growth along the *a* axis to form orientation, which is consistent with SAXS results. Furthermore, the interlayer spacing of the HC-1200 sample is still larger than that of GQD/HC-1000, indicating an improvement in crystallinity by micropressure. Similarly, Raman spectra show that *I_D_*/*I_G_* gradually decreases with increasing pressure and temperature (Fig. S10), revealing the positive correlation between crystallinity and temperature/micropressure.

Then, there is a significant increase in carbon yield under micropressure from 400 to 1,200 °C (Fig. 2D), demonstrating the restriction effect of micropressure on the escape of gaseous molecules. Furthermore, the thermogravimetric analysis (from room temperature to 600 °C with 8 °C min^−1^ in air atmosphere; Fig. 2E) shows that HC-1200 almost completely decomposes at 600 °C, but GQD/HC-1200 increases by 42%, which corresponds to the lower decomposition temperature of amorphous carbon than that of graphite. These analyses reflect that the crystallinity is significantly improved by the thermopressure coupling effect, thus increasing the decomposition temperature.

In combination with the above analysis, a reasonable formation mechanism was proposed based on the thermopressure coupling effect under micropressure (Fig. 2F). First, during the pyrolysis of cellulose, active cellulose is an important intermediate product [31]. The glycosidic bond would disconnect when the temperature reaches about 300 °C, forming the main products such as gaseous levoglucosan and primary coke [32]. During slow cellulose pyrolysis, levoglucosan becomes an important gaseous product [33]. However, large-scale escape of levoglucosan would be restricted by micropressure, and some gaseous volatiles are trapped in the solid products. As the temperature increases, the primary coke is further aromatized and the levoglucosan undergoes secondary pyrolysis [34]. Pyrolysis products mainly include 5-membered ring products, 2-carbon fragments, and tetrose fragments [35]. Under the action of micropressure and heating, those products would be further aromatized to form secondary coke [36]. In conjunction with continuous heating, the degree of graphitization gradually increases for primary and secondary coke. Finally, the primary coke forms HC, and mainly GQDs come from the secondary coke. On the basis of this formation mechanism, the thermopressure coupling effect ensures the integrity of the cellulose skeleton during pyrolysis and improves carbon yield. Meanwhile, GQD is formed and distributed in HC. Consequently, such a thermopressure coupling strategy under micropressure is inspired by the graphite formation process, but homologous carbonaceous heterojunctions are synthesized that are completely different from traditional graphite-like carbon materials.

**Fig. 3.**
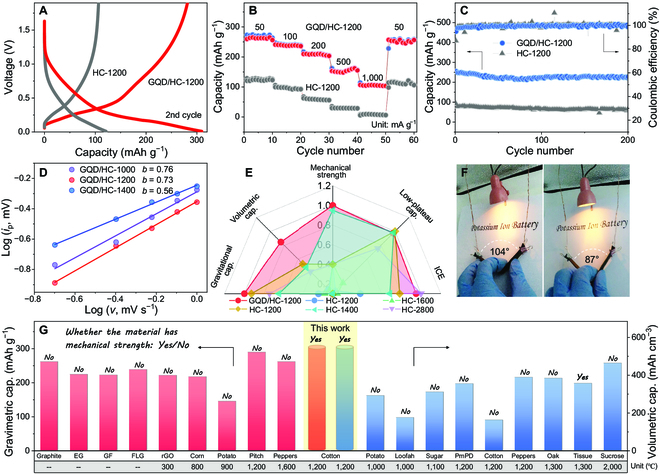
Electrochemical performance. (A) Charge/discharge curves. (B) Rate performances. (C) Cycle performances. (D) *b* values of different samples. (E) Effect of micropressure on the properties of carbon materials (normalized data). (F) Working diagram of the bending fiber battery. (G) Comparison of comprehensive properties among different carbon materials.

The K–ion storage characteristics were then tested in detail. As shown in Fig. 3A, there are 3 stages in the discharge curve of GQD/HC-1200, corresponding to the differential capacity curves (Fig. S11). The sloping region corresponds to the adsorption of K ions in the pores and active sites and the intercalation in the pseudo-graphitic carbon layers [37,38]. The plateau appears when the discharge voltage reaches ~0.3 V, indicating that the electrochemical behavior changes, continue to discharge to ~0.17 V, and the discharge curve changes further. However, HC-1200 shows only 2 features, the turning point of the sloping and plateau region is also ~0.17 V. According to previous research, the region below ~0.17 V corresponds to the intercalation of K ions into graphite-like carbon layers [39]. Therefore, the additional plateau between 0.3 and 0.17 V in GQD/HC-1200 electrode corresponds to the intercalation of K ions between the GQD and HC. At the same time, the total reversible capacity of the HC-1200 electrode is only 120 mAh g^−1^, which is much lower than GQD/HC-1200 (310 mAh g^−1^). Moreover, the ICE of the HC-1200 electrode is only 34%, while GQD/HC-1200 electrode shows 67% (Fig. S12). Because solid electrolyte interface (SEI) films are mainly formed on the surface of electrode materials [40], the low ICE is mainly caused by the high specific surface area (Fig. S13) and structural defects of the HC-1200 electrode. Then, the rate performance of the 2 samples is shown in Fig. 3B. At 1,000 mA g^−1^, the GQD/HC-1200 electrode still maintains 110 mAh g^−1^, while the HC-1200 electrode almost has no capacity. Similarly, the cycling capacity and stability of GQD/HC-1200 are excellent (Fig. 3C).

To fully explore the effect of temperature and micropressure, it is first found that the plateau gradually decreases with the increase of temperature (Fig. S14), corresponding to the change law of crystallization. Then, the influence of different micropressures on the storage performance of K ions was evaluated at 1,200 °C (Fig. S15). The GQD/HC-1200 electrode shows the best capacity and ICE. Furthermore, the overall rate performance is displayed in Fig. S16. In essence, the formation of GQDs is the key to improving the rate performance. The modulation of temperature and micropressure on the crystallinity and defect level optimizes the K–ion storage performance.

The cyclic voltammetry tests were then performed (Fig. S17), and the *b* values representing different electrochemical behaviors were fitted and calculated. As shown in Fig. 3D, the *b* value of GQD/HC-1000 is the largest (0.76), indicating that the pseudocapacitance adsorption behavior is dominant [41]. The *b* value of the GQD/HC-1400 is close to 0.5, which corresponds to the intercalation process dominated by diffusion behavior [42]. On the contrary, the *b* value of GQD/HC-1200 is 0.73, suggesting a joint influence of adsorption and diffusion behavior.

By summarizing the properties of carbon materials (see Table S3 for details), GQD/HC-1200 shows significant advantages compared to most HC without pressure (Fig. 3E). Moreover, the charge/discharge curves among commercial HC, sugar-derived HC, and GQD/HC-1200 were compared (Fig. S18). Although the plateau is low, the capacity of commercial HC anode cannot meet applications. The capacity and plateau of sugar-derived HC are similar to GQD/HC-1200, but sugar-derived HC is a powder material. Therefore, as a high mechanical strength and self-supporting electrode without conductive agent and binder, GQD/HC-1200 has outstanding comprehensive performance. Next, Fig. 3F and Fig. S19 show the working condition of GQD/HC-1200 in different bending states by assembling the electrode material into a fibrous battery. It can easily light the table lamp (working voltage of 2.4 V). The flexibility and high mechanical strength ensure that the material can maintain structural integrity during bending, thus showing stable performance and expanding the application of carbon materials.

More significantly, GQD/HC-1200 shows a significant improvement in compactness. The volume per milligram of GQD/HC-1200 is ~0.03 cm^3^, while the volume per milligram is 0.07 cm^3^ for HC-1200. Therefore, the volumetric and gravimetric capacity of K–ion storage was further studied based on previously reported data (as shown in Fig. 3G; data sources are listed in Table S4). For gravimetric capacity, GQD/HC-1200 exhibits an advantage compared to graphite-like carbon materials (graphite, expanded graphite, few-layer graphene, and graphite foam) and non-graphite carbon materials (reduced graphene oxide, soft carbon, and HC), which originates from the synergistic effect of the heterostructure. The volumetric capacity of GQD/HC-1200 (558 mAh cm^−3^) is greater than pressure-free samples reported in the literature. The sample obtained by thermal and micropressure synergy is superior to those prepared by the ordinary carbonization method in terms of volumetric capacity, plateau, mechanical strength, etc., suggesting the practicability of this strategy.

**Fig. 4.**
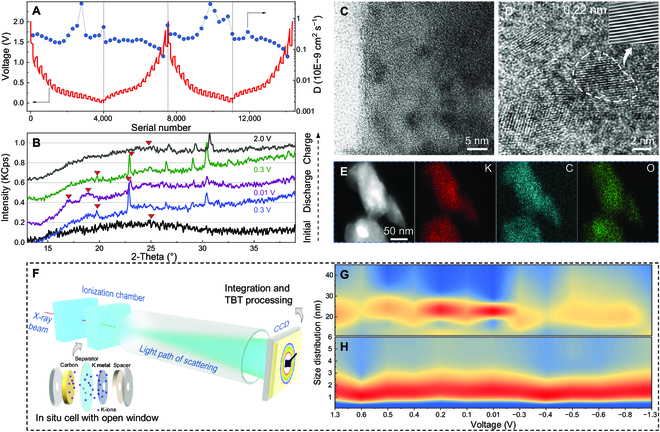
Analysis of the potassium storage mechanism. (A) GITT result of GQD/HC-1200. (B) Ex situ XRD results of GQD/HC-1200. (C to E) HRTEM and element mapping images of GQD/HC-1200 after discharging to 0.01 V. (F) Schematic diagram of in situ synchrotron SAXS experiment. (G and H) Integration results in different sizes under different states of discharge/charge.

Next, GITT was performed on the GQD/HC-1200 electrode to explore the K–ion storage process (Fig. 4A). As the discharge proceeds, the diffusion coefficient of K ions gradually decreases, corresponding to a gradual transition from surface adsorption to interlayer intercalation. However, when the voltage reaches ~0.3 V, the diffusion coefficient increases significantly and then decreases at ~0.17 V, indicating that the storage behavior of K ions has changed significantly. Considering the charge/discharge curves, GITT also shows that the plateau at ~0.17 to 0.30 V is associated with GQD. Therefore, the mechanism is reasonably conjectured: A significant increase in the diffusion coefficient at ~0.17 to 0.3 V is due to the storage of K ions in the interlayers between GQD and HC. After further discharge, the region below ~0.17 V corresponds to the K–ion intercalation into graphite-like carbon layers of HC. As a result of the large size of the carbon layers, the diffusion coefficient of the K ions decreases significantly. A similar phenomenon is observed in the subsequent cycle, indicating that the process has good reversibility. In addition, the GITT results of other samples (Fig. S20) imply that the appropriate carbonization temperature plays a decisive role in improving the K–ion storage performance.

Concerning structural changes, ex situ XRD patterns indicate that the diffraction peak of the original GQD/HC-1200 gradually shifts to 22.90° and 19.75° when discharged to 0.3 V (the red arrows in Fig. 4B), corresponding to stage 3 and stage 2 graphite intercalation compounds (GICs), respectively [43]. After discharge to 0.01 V, the interlayer spacing expands to 5.24 Å (16.9°; stage 1 K-GICs), due to intercalation related to GQD and graphite-like carbon layers. Unlike graphite, the diffraction peaks of stage 2 (18.89°) and stage 3 (23.01°) still exist in a fully discharged state, indicating that the complete phase transition would not occur in HC, and K ions could be stored stably in some carbon layers with large interlayer spacing. During charging, the main diffraction peaks change inversely and revert to the initial state after charging to 2.0 V, indicating that the process is highly reversible. Subsequently, the TEM photos also show the GQD and HC structure when discharging to 0.01 V. Figure 4C shows that the microstructure of the GQD/HC-1200 sample has not changed significantly after discharge and that the GQD is stable. After further magnification, there are obvious lattice fingers of 0.22 nm in GQD (Fig. 4D), which is mainly consistent with the initial state. Then, the element mapping images at 0.01 V show that C, O, and K elements are evenly distributed (Fig. 4E), also indicating good structural uniformity.

Furthermore, in situ synchrotron SAXS analyzed the pore structure in the GQD/HC-1200 electrode. As shown in Fig. 4F, the constructed in situ device is mainly composed of an optical path system and a perforated battery (polyimide film window). Synchrotron x-rays are irradiated into the electrode material and then scattered. The scattered signal is collected by charge-coupled device (CCD), and the scattered data are obtained through integral processing. First, Fig. 4G shows that the mesopore size has slight irreversible changes in the charge/discharge process. The peak intensity at ~24 nm increases when discharging to 0.01 V, and the mesopore slightly expands during the charging process (the value of the charge voltage is negative on the lateral axis). Similar to previous reports, this phenomenon originates from the slight deformation of the overall structure caused by K–ion intercalation [37]. Next, in Fig. 4H, the decrease of size distribution during 1.3 to 0.6 V (discharge) corresponds to the adsorption of K ions in the surface micropores [44]. Then, as the intercalation of the K ions (0.6 to 0.01 V in the discharge process), the internal micropore size distribution is enlarged from 1.1 to ~1.8 nm due to volume expansion [45]. When charging to 0.4 V, the size distribution becomes significantly small. The micropore size returns to the initial level after further charging to 1.3 V, indicating a reversible structure change. The results of the in situ synchrotron SAXS show that the pore structure of the GQD/HC-1200 does not change significantly during the charge/discharge process, suggesting that the unique heterojunction is conducive to improving the stability of the electrode material.

**Fig. 5.**
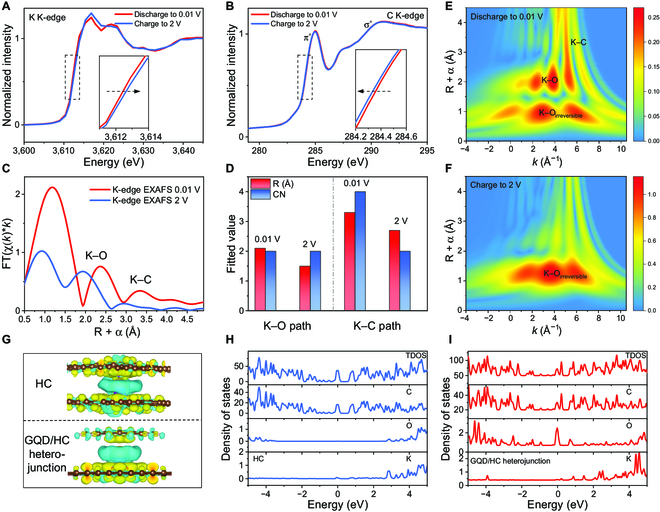
Insight into the K–ion storage mechanism by synchrotron x-ray absorption spectra and DFT calculation. (A and B) XANES of the K and C elements, respectively. (C) *R*-space data based on EXAFS of K element. (D) Fitting results of the *R*-space. (E and F) WT contour map of discharge/charge states. (G) The differential charge density of HC and GQD/HC heterojunction. (H and I) DOS spectra of 2 models. FT, Fourier transform.

For exploring the storage mechanism, the synchrotron x-ray absorption near-edge structure (XANES) and expanded x-ray absorption fine structure (EXAFS) were analyzed for GQD/HC-1200 in different charge/discharge states (the spectra were processed by Athena software [46]). As shown in Fig. 5A, when discharging to 0.01 V, the absorption edge shifts to low energy by 0.3 eV compared to that in 2 V (charged state). The decrease of the absorption edge energy indicates that the electron shifts to K ions during the formation of K-GICs, thus enhancing the reduction state of K ions. After charging to 2 V, the K ions are deintercalated, leading to an upward trend in absorption edge energy. Because of the presence of an irreversible K–ion store, the absorption edge changes only slightly. In contrast to the absorption edge of the K element, as shown in Fig. 5B, the absorption edge energy of the C element increases when discharging to 0.01 V, indicating that the electrons shift toward the K ions in the discharged state. When charging to 2 V, the electrons return to the C element because of the deintercalation of the K ions, which reduces the absorption edge energy and shows a reduction state of the C element.

The XANES of the O element also shows the electron transfer during the process of charge/discharge. As shown in Fig. S21, there are significant π* and σ* excitations [47]. Then, the absorption edge shifts from the initial 532.0 to 533.1 eV after discharging to 0.01 V, indicating that the oxidation state of the O element increases, caused by the adsorption of the K ions. After charging to 2 V, the absorption edge partially returns to the initial state (532.4 eV). The XANES results show that the O-containing functional groups are mainly electron donor groups, which also complements the relevant research [48,49]. The electrons are transferred to the adsorbed K ions, so the oxidation state of the O element is significantly increased. There are still some irreversible O adsorption behaviors, such as disappeared π* excitation, resulting in capacity loss.

Next, the EXAFS of the K element is analyzed and fitted (Fig. S2). For the *R*-space data of 0.01 V (discharge) and 2 V (charge), the K−O path is mainly from the adsorption of K ions on oxygen-containing functional groups, and the K−C path is from the K-GICs. In Fig. 5C, it is found that both the bond length and the relative intensity at 0.01 V are larger than that at 2 V. After further fitting, as shown in Fig. 5D, the bond length of the K−O path is 2.1 Å, and the coordination number is 2. The bond length of the K−C path is 3.3 Å, and the coordination number is 4. Compared to the K−C bond of 3.12 Å in graphite [50], the K−C bond length increases in GQD/HC-1200 with expanded interlayer spacing. These indicate the “loose” existence state of K ions in carbon layers, which helps to improve the rapid (de)intercalation ability of K ions with a large ion radius. After charging to 2 V, the coordination number and bond length of the K−C path decrease, indicating the reversible deintercalation of K ions. The bond length of the K−O path decreases because of desorption of K ions at the oxygen site, while the unchanged coordination number is caused by the stable formation of the SEI. Additionally, to intuitively distinguish the information of the *k* and *R*-space, wavelet transform (WT) was used to analyze the EXAFS data. As shown in Fig. 5E, the WT contour map of GQD/HC-1200 at 0.01 V (discharge) shows 3 regions with high-intensity distribution, of which 2.1 Å (*k* = 3.6 Å^−1^) is attributed to the K−O coordination. The K−C coordination is widely distributed, indicating K-GICs in different stages. Then, the coordination intensities of K−O and K−C paths after charging to 2 V disappeared or decreased (Fig. 5F), suggesting that a reversible reaction occurs. The intensity distribution in *R* = ~1 Å of the 2 states may come from the irreversible adsorption of the K−O path and SEI.

Then, electronic structure changes of the GQD/HC heterojunction in the process of K–ion storage were explored by density functional theory (DFT) calculation. Figure S23 and Fig. 5G show the model and differential charge density of pure HC and GQD/HC heterojunction. Both models contain O sites, providing a comparison similar to that of the actual situation. First, the centers of the 2 models are K ion. Yellow represents the area where the charge density increases and light blue represents the decrease. The charge density distribution is uniform for the K ion between the carbon layers of pure HC; both carbon layers have a limiting effect on the K ion. As for the GQD/HC heterojunction, the charge density is higher in the carbon layer of HC than that in GQD, which may be rooted in the size and crystallinity of GQD. Consequently, the charge distribution tends to the HC layer. The asymmetric distribution of charge density between the GQD and HC is more conducive to the electron transfer between the K ions and HC, thereby improving the high-rate dynamics.

Then, as shown in Fig. 5H and I, the calculation results of the density of states (DOS) show that the GQD/HC heterojunction has one more peak than HC at the Fermi level in the total DOS spectrum, indicating more electronic states near the Fermi level. Hence, the conductivity is enhanced in the GQD/HC heterojunction, which helps to realize fast electron transfer. This difference is further proved by partial DOS. In the HC model, the partial DOS data of C and K show only one peak at the Fermi level, while there is no peak for the O element. Considering irreversible peak changes in XANES of O, the O element does not play a positive role during K–ion storage. However, the GQD/HC heterojunction model shows obvious differences. There are many peaks near the Fermi level of C, K, and O elements, in which the extra electronic states at the Fermi level come from the asymmetric distribution of GQD and HC with high disorder. Therefore, combined with the electrochemical properties and K–ion storage mechanism, it has been proved that the K ions can be effectively stored in interlayers between GQD and HC. Because GQD changes the local electronic structure, fast K–ion (de)intercalation and charge transfer are more favorable than those of pure HC, eventually improving the comprehensive performance of the GQD/HC heterojunction.

## Conclusion

Inspired by the formation mechanism of natural graphite, a thermopressure coupling strategy under micropressure (<400 Pa) was applied to prepare carbon materials. A novel thermopressure response was examined using various characterization methods. Homologous GQD/HC heterojunctions were synthesized under this strategy. Adjusting the temperature and pressure, the crystallinity was regulated and the heterostructures had obvious mechanical strength (0.07 N mm^−1^). The remarkable mechanical strength of the heterojunction is an uncommon characteristic based on the high-temperature preparation method. When used in KIBs, GQD/HC-1200 showed high volumetric capacity (558 mAh cm^−3^), gravimetric capacity (310 mAh g^−1^), and ICE of 67%, while the capacity and ICE of the direct carbonization product were only 120 mAh g^−1^ and 34%, respectively. The total capacity of GQD/HC-1200 comes from the pore structure, GQD, and HC. The results from XANES and EXAFS illustrate the electron transfer and coordination environment in K-GICs. The changes in electronic states in the GQD/HC heterojunction were analyzed by DFT calculations and proved the conjecture that K ions can be effectively (de)intercalated in interlayers between GQDs and HC. This work has completed functional mimicry and improvement, and a thermopressure coupling strategy under micropressure injects new vitality into material design and industrialization.

## Materials and Methods

### Material synthesis

Commercial pure cotton was used as the raw material; its chemical composition is cellulose. Cotton was sandwiched between 2 graphite plates for heat treatment at different temperatures in the tubular furnace (as shown in Fig. S1). The length and width of the graphite plates are fixed at 100 × 40 mm, and the thickness is variable to change the weight and then adjust the micropressure value. Under 181.4 Pa, the products prepared at 1,000, 1,200, and 1,400 °C were marked as GQD/HC-1000, GQD/HC-1200, and GQD/HC-1400, respectively. Cotton was directly heated to 1,200 °C to prepare the contrast sample without micropressure (0 Pa, marked HC-1200). For all cases, the heating/cooling rate is 2 °C min^−1^, the holding time is 1 h, and the argon flow rate is 30 ml min^−1^. Other specific conditions were described elsewhere.

### Material characterizations

X-ray diffraction (XRD) was performed on the Bruker D8 Advance x-ray diffractometer (Cu Kα radiation, λ = 1.54178 Å), and Raman spectra were measured on the Renishaw Invia device (532 nm). Morphology was demonstrated using the transmission electron microscope (TEM; FEI Tecnai G2 F20 S-TWIN), and the field-emission scanning electron microscope (SEM; Hitachi S-4800). The tensile strength test was completed on the universal material testing machine (1036PC, Taiwan POOTAB); the tested sample has a size of 80 × 30 × 0.4 mm. The N_2_ adsorption–desorption apparatus (ASAP2460, Micromeritics Instruments) was used to characterize the specific surface area and pore properties. Thermogravimetric analysis was completed with the thermal analyzer (TGA 55, TA Instruments). Synchrotron small-angle x-ray scattering (SAXS) tests were performed at the Beijing Synchrotron Radiation 1W2A Work Station. Synchrotron x-ray absorption spectra were obtained from the Beijing Synchrotron Radiation 4B7A and 4B7B Work Station.

### Electrochemical measurements

All electrochemical tests were performed in coin cells (CR2032), and the materials were directly used as free-standing electrodes. The areal mass loading of GQD/HC-1200 is ~1.3 mg cm^−2^. The glass fiber (Whatman GF/A) and K metal were used as separators and counter/reference electrodes, respectively. The electrolyte was the solution of 0.8 M KPF_6_ dissolved in a mixture of ethylene carbonate and diethyl carbonate (volume ratio of 1:1). Subsequently, the cells were assembled in the glove box filled with argon atmosphere (Mikrouna; O_2_ and H_2_O < 0.1 ppm). Galvanostatic charge/discharge cycles and galvanostatic intermittent titration technique (GITT) tests were conducted on the Neware battery test system (CT-3008) within the voltage range of 0.01 to 2.0 V (versus K^+^/K). Cyclic voltammetry tests were carried out on the CH Instruments electrochemical workstation (CHI660D) with scan rates of 0.2 to 1.0 mV s^−1^. For the GITT tests, the cell was discharged/charged at 50 mA g^−1^ with a current pulse duration and an interval time of 20 min.

## Data Availability

All of the relevant data in this work are available upon reasonable request from the corresponding author.
