# Supplementary material for: Thermopressure Coupling Effect Mimicking Natural Graphite Formation to Enhance the Storage K–Ion Performance of Carbonaceous Heterostructures
Source: Research (Wash D C). 2023 Mar 27;6:0092. doi: 10.34133/research.0092 (PMC10045921; doi:10.34133/research.0092)
Supplement: Supplementary 1 — Figs. S1 to S23 Tables S1 to S4 [file research.0092.f1.pdf]

Thermopressure Coupling Effect Mimicking Natural Graphite Formation to Enhance the Storage K-ion Performance of Carbonaceous Heterostructures

Tianyi Ji[1], Xiaoxu Liu[1,*], Hui Wang[2], Yunli Shi[1], Yang Li[1], Man Zhang[1], Junqi Li[1], Hui Liu[1], Ze Xiang Shen[2,*]

[1] Shaanxi Key Laboratory of Green Preparation and Functionalization for Inorganic Materials, School of Material Science and Engineering, Shaanxi University of Science and Technology, Xi'an 710021, Shaanxi, China

[2] Division of Physics and Applied Physics, School of Physical and Mathematical Sciences, Nanyang Technological University, Singapore 637371, Singapore

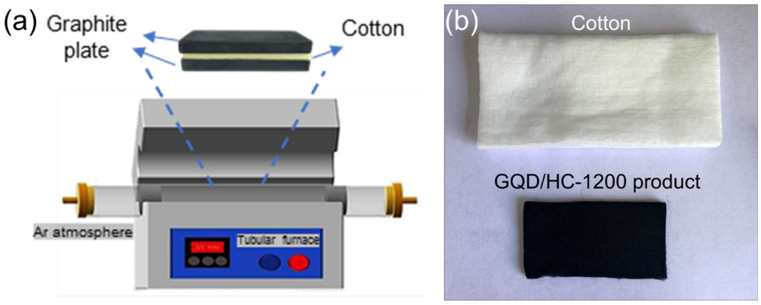

Fig. S1 (a) Schematic illustration of preparation process; (b) Photos of product macrostructure

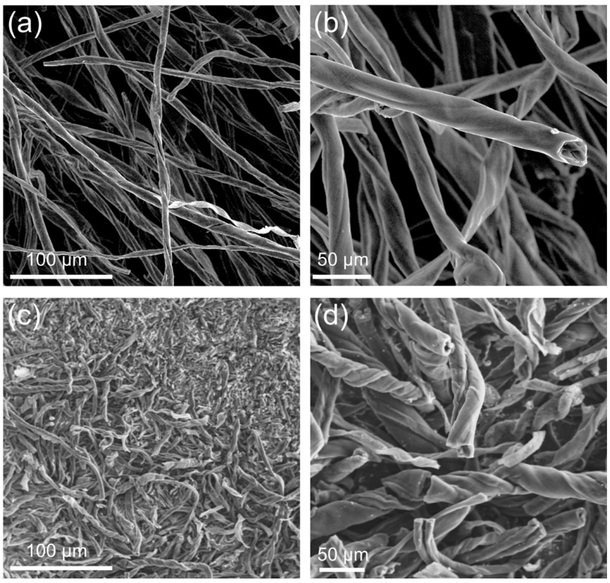

Fig. S2 SEM images. (a, b) GQD/HC-1200; (c, d) HC-1200

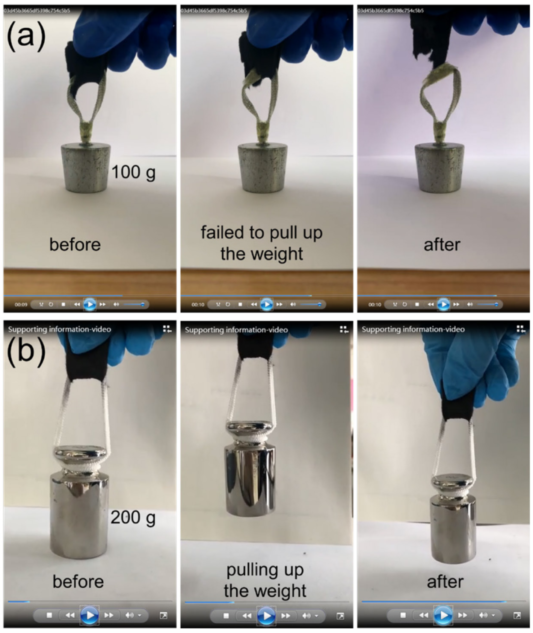

Fig. S3 Screenshots in the video of pulling up the weight. (a) HC-1200; (b) GQD/HC-1200

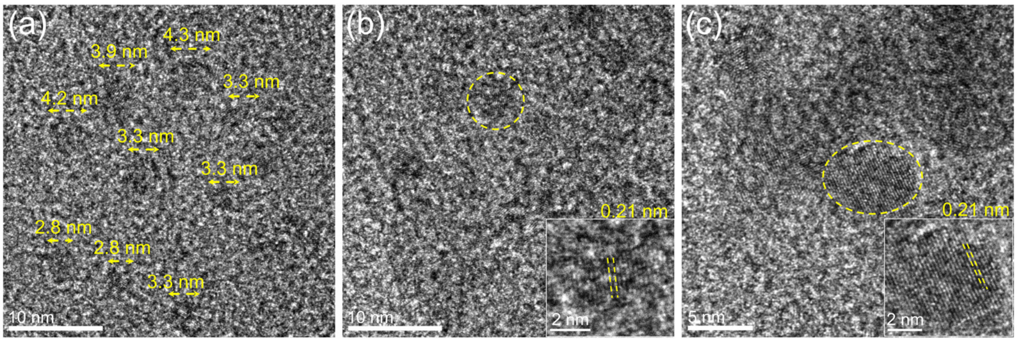

Fig. S4 TEM images of GQD/HC-1200

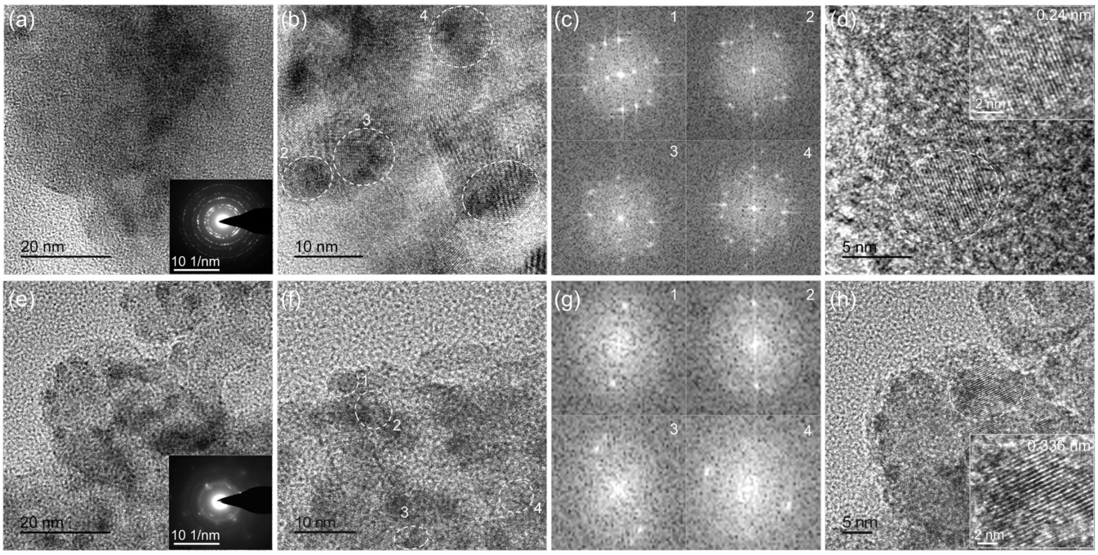

Fig. S5 TEM images. (a-d) GQD/HC-1000; (e-h) GQD/HC-1400

The synergistic effect was explored for temperature and micropressure. Under 1000°C and 181.4 Pa, the sample shows an irregularly stacked structure, with diffraction rings and dispersed diffraction spots in the SAED pattern (Fig. S5a), indicating that the crystallinity of GQD is relatively poor at 1000°C. Some regions show obvious moiré fringes (Fig. S5b), indicating that the heterojunction has been formed at 1000°C with micropressure. Further, Fig. S5c, d exhibit that the GQDs have visible diffraction spots and a lattice spacing of 0.24 nm[1]. Next, the SAED and FFT patterns also show regular diffraction spots in the sample of 1400°C and 181.4 Pa (Fig. S5e-g). Furthermore, the lattice spacing of GQD is 0.336 nm (Fig. S5h), which is close to the (002) crystal plane of graphite, indicating that the crystallinity of GQD gradually increases with the rising temperature.

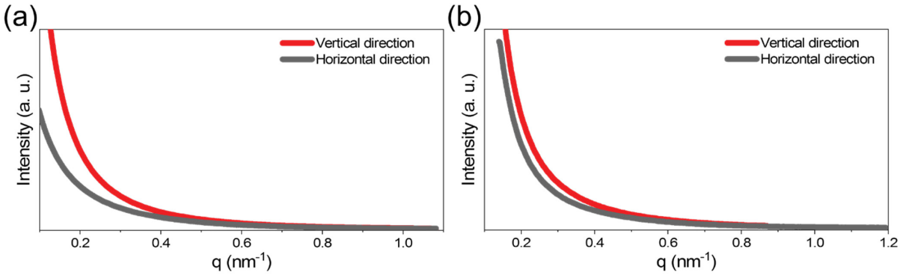

Fig. S6 Data of SAXS intensity *vs.* scattering vector length q obtained by Fit_2D software. (a) GQD/HC-1200; (b) HC-1200

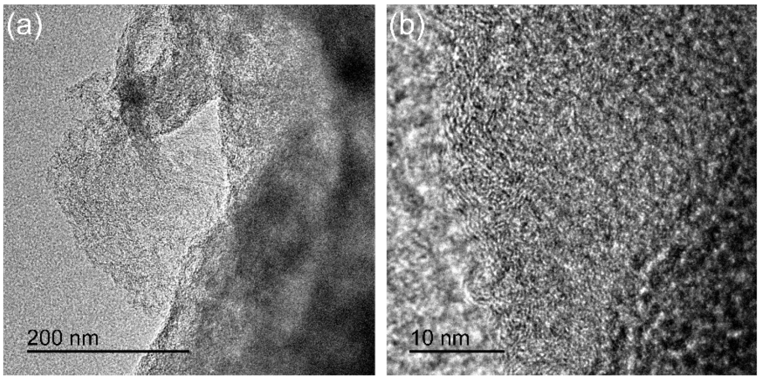

Fig. S7 TEM images of HC-1200

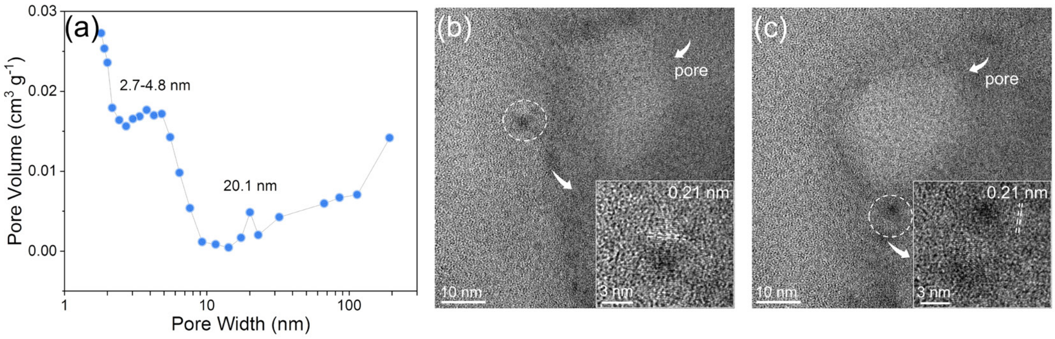

Fig. S8 Pore size analysis based on N_2 adsorption-desorption result and TEM images of GQD/HC-1200

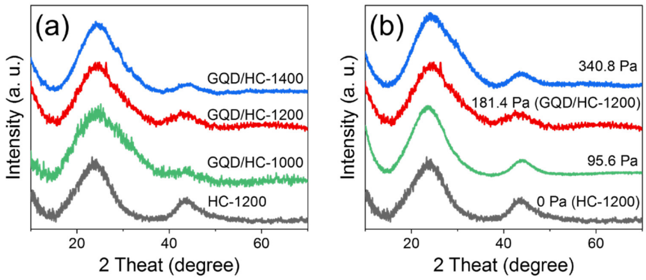

Fig. S9 XRD results of different samples

Table S1 Interlayer spacing and grain size under different temperatures

	d_{002} (nm)	L_a (nm)	L_c (nm)
GQD/HC-1400	3.61	6.07	1.09
GQD/HC-1200	3.69	5.25	1.03
GQD/HC-1000	3.78	4.51	0.98
HC-1200	3.79	4.52	1.19

Table S2 Interlayer spacing and grain size under different pressures at 1200°C

	d_{002} (Å)	L_a (nm)	L_c (nm)
340.8 Pa	3.66	5.30	0.93
181.4 Pa	3.69	5.25	1.03
95.6 Pa	3.77	5.01	1.15
0 Pa	3.79	4.52	1.19

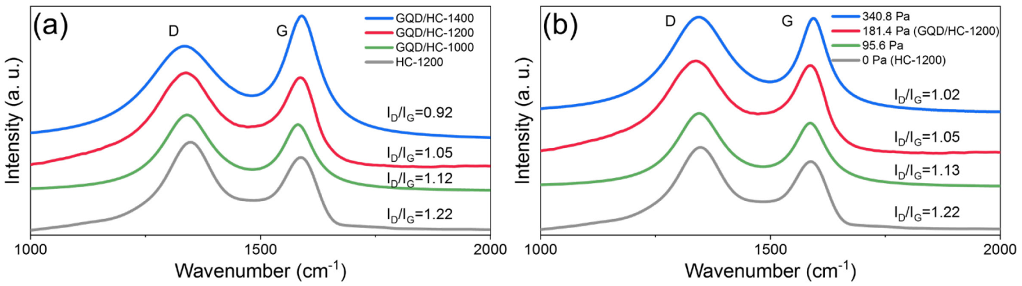

Fig. S10 Raman results of different samples

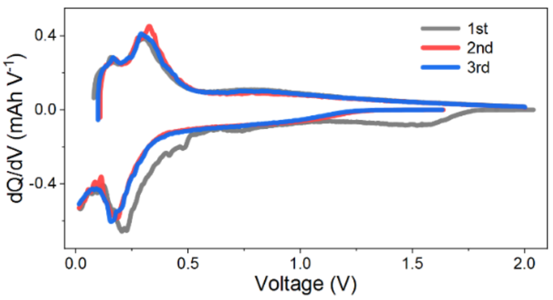

Fig. S11 Differential capacity analysis of charge/discharge curves

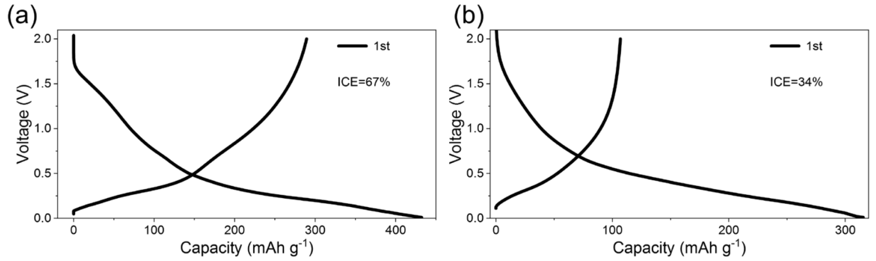

Fig. S12 Charge/discharge curves in 1st cycle. (a) GQD/HC-1200; (b) HC-1200

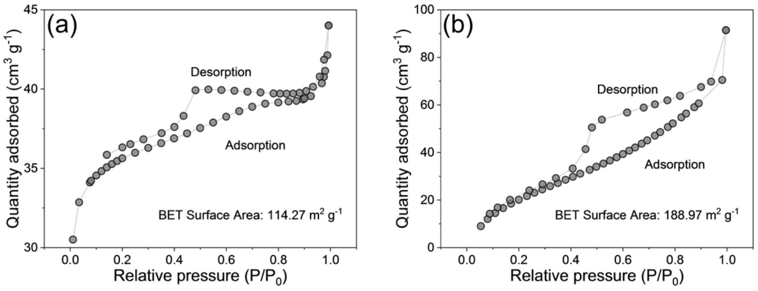

Fig. S13 N_2 adsorption-desorption curves. (a) GQD/HC-1200; (b) HC-1200

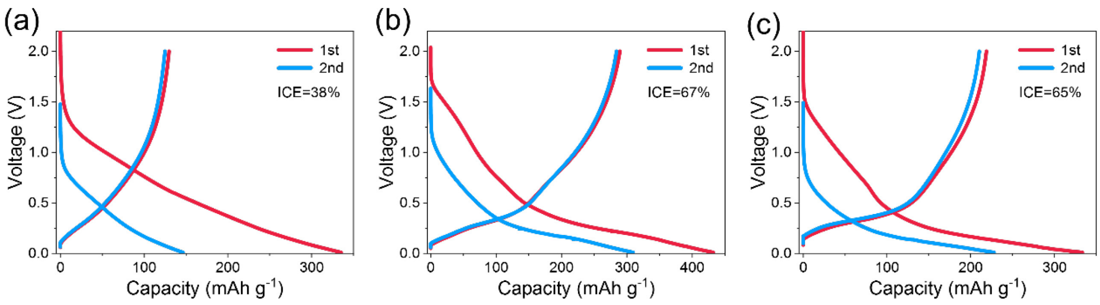

Fig. S14 Change/discharge curves under different temperatures. (a) GQD/HC-1000; (b) GQD/HC-1200; (c) GQD/HC-1400

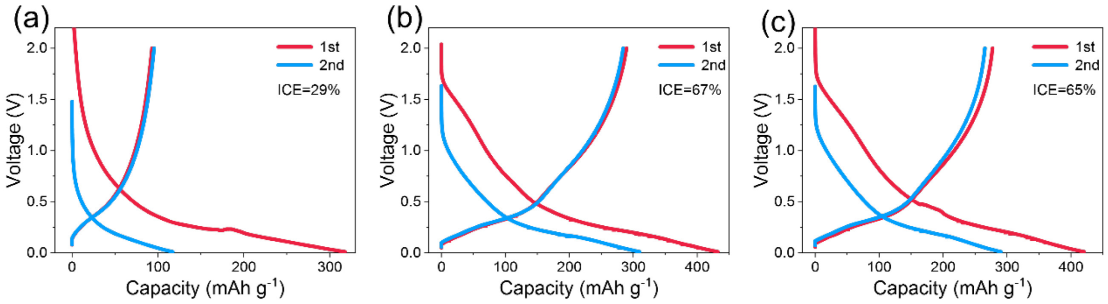

Fig. S15 Change/discharge curves under different pressure based on 1200℃. (a) 95.6 Pa; (b) 181.4 Pa (GQD/HC-1200); (c) 340.8 Pa

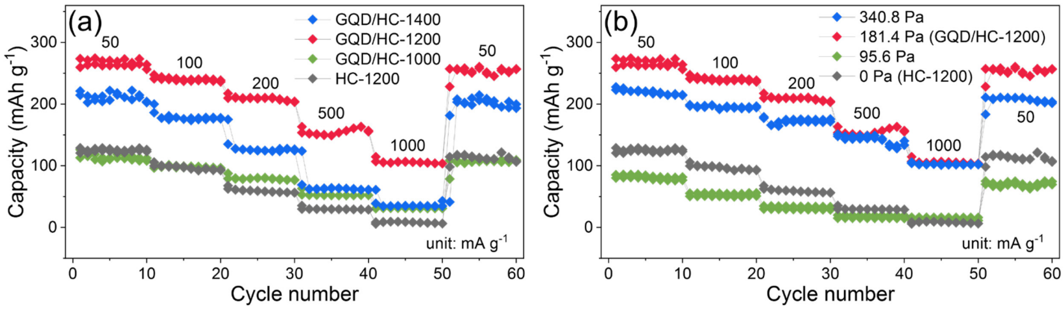

Fig. S16 Rate performance of samples under different temperatures and pressures

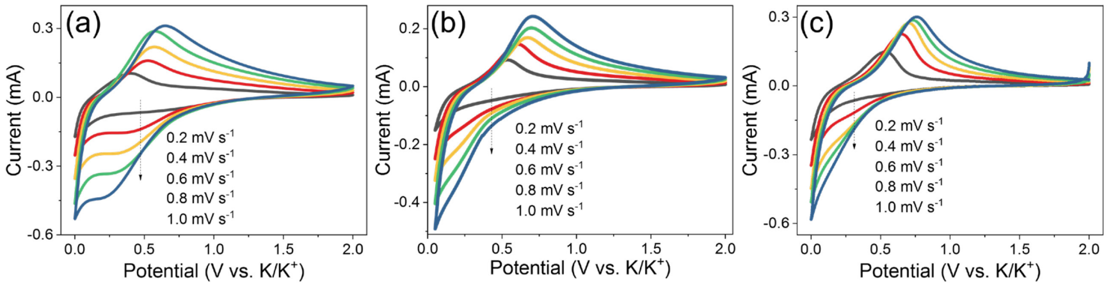

Fig. S17 CV curves of different samples. (a) GQD/HC-1000; (b) GQD/HC-1200; (c) GQD/HC-1400

Table S3 Comparison of properties among GQD/HC-1200 and hard carbon

	**Mechanical strength**	**Volumetric cap. (mAh cm^{-3})**	**Gravitational cap. (mAh g^{-1})**	**Low-plateau cap. (mAh g^{-1})**	**ICE (%)**
GQD/HC-1200 [a]	yes	558	310	196	67
HC-1200 [a]	no	166	121	71	33
HC-1600 [a]	no	157	105	88	19
HC-1200 [b]	no	337	246	163	70
HC-1400 [b]	no	409	292	200	57
HC-2800 [b]	yes	377	222	197	51

[a] means the data comes from this work. [b] represents the data from Ref. [2-4] in turn. The volumetric capacity was estimated according to the material real density (referenced to Chinese Patent 01807830.3) and gravitational capacity. Under the actual volume of products, the density of GQD/HC-1200 was estimated to be 1.4 times that of HC-1200.

Table S4 Data sources in Fig. 3g

Materials	Sources
Graphite	J. Am. Chem. Soc., 2015, 137, 11566-11569[5]
Expanded graphite	Chem. Eng. J., 2020, 381, 122677[6]
Graphite foam	Adv. Energy Mater., 2019, 9, 1900579[7]
Few-layer graphene	Nanoscale, 2016, 8, 16435-16439[8]
rGO-300	Nano Lett., 2015, 15, 7671-7677[9]
Corn-800	Electrochim. Acta, 2019, 324, 134902[10]
Potato-900	Electrochim. Acta, 2019, 293, 364-370[11]
Pitch-1200	Adv. Mater., 2020, 32, 2000505[12]
Peppers-1600	J. Power Sources, 2019, 444, 227310[3]
Potato-1000	Electrochim. Acta, 2019, 293, 364-370[11]
Loofah-1000	Electrochim. Acta, 2019, 306, 446-453[13]
Sugar-1100	Adv. Energy Mater., 2016, 6, 1501874[14]
PmPD-1200	ACS Appl. Mater. Interfaces, 2020, 12 (11), 13182-13188[15]
Peppers-1200	J. Power Sources, 2019, 444, 227310[3]
Oak-1300	Chem. Eng. J., 2020, 391, 123576[16]
Tissue-1300	Energy Environ. Sci., 2022, 15, 158-168[17]
Sucrose-2000	ACS Nano, 2019, 13, 10631-10642[18]

the calculation method of volumetric capacity is the same as that in Fig. 3e.

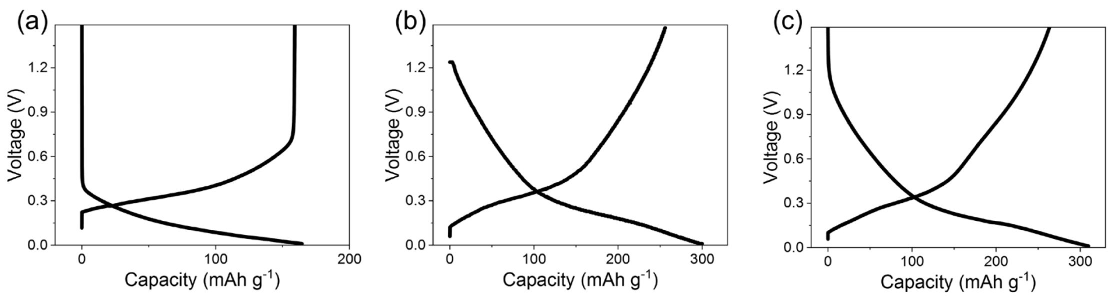

Fig. S18 Charge/discharge curves in 2nd cycle under 30 mA g^{-1}. (a) commercial hard carbon; (b) sugar-derived hard carbon[14]; (c) GQD/HC-1200 in this work

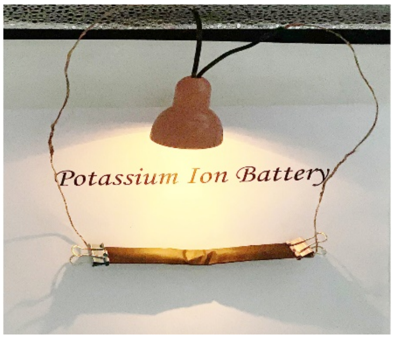

Fig. S19 Working condition of fiber battery in the initial state

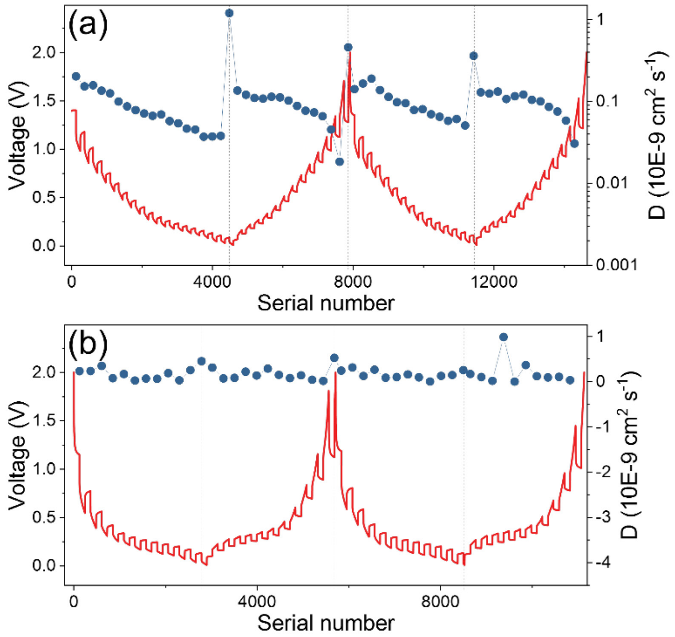

Fig. S20 GITT tests. (a) GQD/HC-1000; (b) GQD/HC-1400

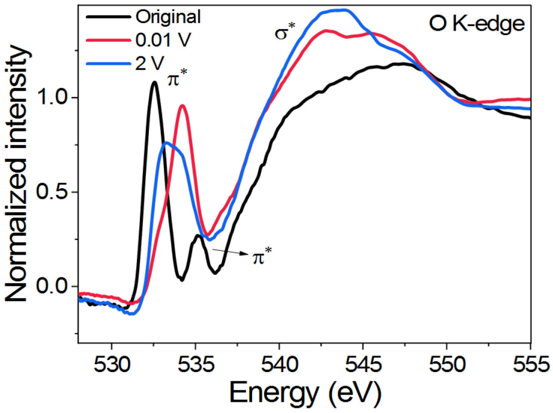

Fig. S21 XANES of O element in GQD/HC-1200

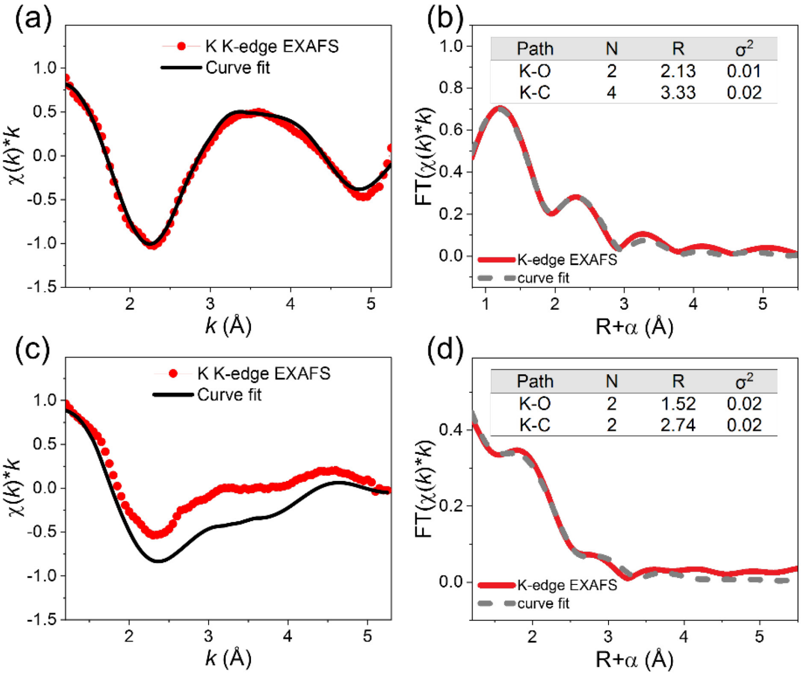

Fig. S22 Data analysis of EXAFS of K element. (a, b) 0.01 V; (c, d) 2 V

For two fits, S_0^2 was guessed to be 1, and the number of variable parameters was 8, out of a total of 12.1 independent data points. The paths were reasonably inferred and fitted according to the related compounds, as well as the set value of the coordination number. ΔE_0 was refined as a global fit parameter, returning values of (0 ± 1) eV for EXAFS data of 2 V, and (-9 ± 3) eV for EXAFS data of 0.01 V. Data ranges of 2 V: 1.0 $\leq$ k $\leq$ 5.5 $Å^{-1}$, 1.2 $\leq$ R $\leq$ 5.5 Å. The R factor for this fit was 1.4%. For EXAFS data of 0.01 V, data ranges: 1.3 $\leq$ k $\leq$ 5.5 $Å^{-1}$, 0.8 $\leq$ R $\leq$ 5.5 Å. R factor for this fit was 1.6%.

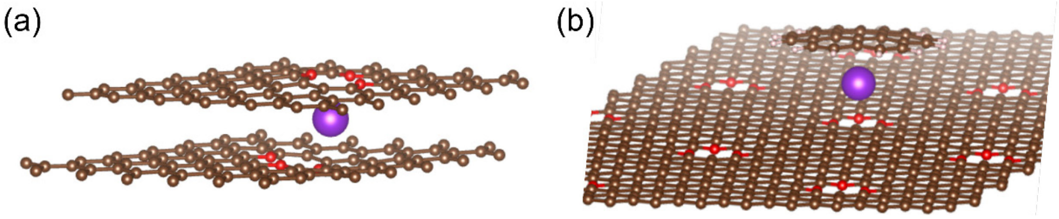

Fig. S23 DFT calculation models (brown represents carbon, purple for potassium, and red for oxygen). (a) hard carbon; (b) GQD/HC heterojunction

The calculations were conducted by using density functional theory (DFT) implemented in Vienna Ab initio Simulation Package (VASP). The projector augmented wave (PAW) method was used with a kinetic energy cutoff of 300 eV, and the convergence criteria of 10^{-5} eV for the total energy and 0.03 eV $Å^{-1}$ for atomic forces were used.

References

1. J. Peng, W. Gao, B. K. Gupta, Z. Liu, R. Romero-Aburto, L. Ge, L. Song, L. B. Alemany, X. Zhan, G. Gao, S. A. Vithayathil, B. A. Kaipparettu, A. A. Marti, T. Hayashi, J.-J. Zhu and P. M. Ajayan, *Nano Lett.*, 2012, **12**, 844-849.
2. X. He, J. Liao, Z. Tang, L. Xiao, X. Ding, Q. Hu, Z. Wen and C. Chen, *J. Power Sources*, 2018, **396**, 533-541.
3. C. Chen, M. Wu, Y. Wang and K. Zaghib, *J. Power Sources*, 2019, **444**, 227310.
4. X. Lin, J. Huang and B. Zhang, *Carbon*, 2019, **143**, 138-146.
5. Z. Jian, W. Luo and X. Ji, *J. Am. Chem. Soc.*, 2015, **137**, 11566-11569.
6. Z. Sang, D. Su, J. Wang, Y. Liu and H. Ji, *Chem. Eng. J.*, 2020, **381**, 122677.
7. J. Liu, T. Yin, B. Tian, B. Zhang, C. Qian, Z. Wang, L. Zhang, P. Liang, Z. Chen and J. Yan, *Adv. Energy Mater.*, 2019, **9**, 1900579.
8. K. Share, A. P. Cohn, R. E. Carter and C. L. Pint, *Nanoscale*, 2016, **8**, 16435-16439.
9. W. Luo, J. Wan, B. Ozdemir, W. Bao, Y. Chen, J. Dai, H. Lin, Y. Xu, F. Gu, V. Barone and L. Hu, *Nano Lett.*, 2015, **15**, 7671-7677.
10. Q. Wang, C. Gao, W. Zhang, S. Luo, M. Zhou, Y. Liu, R. Liu, Y. Zhang, Z. Wang and A. Hao, *Electrochim. Acta*, 2019, **324**, 134902.
11. W. Cao, E. Zhang, J. Wang, Z. Liu, J. Ge, X. Yu, H. Yang and B. Lu, *Electrochim. Acta*, 2019, **293**, 364-370.
12. Y. Liu, Y.-X. Lu, Y.-S. Xu, Q.-S. Meng, J.-C. Gao, Y.-G. Sun, Y.-S. Hu, B.-B. Chang, C.-T. Liu and A.-M. Cao, *Adv. Mater.*, 2020, **32**, 2000505.
13. Z. Wu, L. Wang, J. Huang, J. Zou, S. Chen, H. Cheng, C. Jiang, P. Gao and X. Niu, *Electrochim. Acta*, 2019, **306**, 446-453.
14. Z. Jian, Z. Xing, C. Bommier, Z. Li and X. Ji, *Adv. Energy Mater.*, 2016, **6**, 1501874.
15. X.-S. Tao, Y.-G. Sun, Y. Liu, B.-B. Chang, C.-T. Liu, Y.-S. Xu, X.-C. Yang and A.-M. Cao, *ACS Appl. Mater. Interfaces*, 2020, **12**, 13182-13188.
16. S. Alvin, C. Chandra and J. Kim, *Chem. Eng. J.*, 2020, **391**, 123576.
17. T. Zhang, Z. Mao, X. Shi, J. Jin, B. He, R. Wang, Y. Gong and H. Wang, *Energy*

Environ. Sci., 2022, **15**, 158-168.
18. Z. Liu, J. Wang, X. Jia, W. Li, Q. Zhang, L. Fan, H. Ding, H. Yang, X. Yu and X. Li, *ACS Nano*, 2019, **13**, 10631-10642.
